# Recurrent genetic alterations in hepatitis C-associated hepatocellular carcinoma detected by genomic microarray: a genetic, clinical and pathological correlation study

**DOI:** 10.1186/s13039-014-0081-8

**Published:** 2014-11-25

**Authors:** Yajuan J Liu, Yang Zhou, Matthew M Yeh

**Affiliations:** Department of Pathology, University of Washington, 1959 NE Pacific Street, Box 357470, Seattle, WA 98195 USA

**Keywords:** Hepatocellular carcinoma, Hepatitis-C virus, Copy number aberration, Copy number variants, Genomic microarray, Prognosis, Pathological correlation

## Abstract

**Background:**

In the US, approximately 50% of hepatocellular carcinoma (HCC) is caused by hepatitis-C virus (HCV) infection. The molecular mechanism of a malignant transformation of hepatocyte induced by HCV infection is still largely unclear. There are several clinical and pathological staging systems for HCC, but none of them include biological parameters as predictors for prognosis and there has not been a standardized molecular classification of HCC. To understand the underlying pathogenic genetic alterations in HCV-associated HCC and aid in molecular classification of HCC and patient prognosis, microarray analysis of DNA copy number alterations in HCC were conducted using whole genome microarray with DNA from formalin-fixed paraffin-embedded (FFPE) specimens of both cancer tissues and paired nearby cirrhotic non-neoplastic tissues.

**Results:**

Our results show that the most common chromosomal aberrations (>5 Mb) observed in HCC were chromosomal gains of 1q (80%), 8q (60%), 7q (40%), 5p (33%), 7p (33%), Xq (33%), 5q (27%), and Xp (20%), as well as chromosome losses of 17p (40%), 4q21.21-q26 (33%), 8p (33%), 1p36.11-pter (20%), and 9p (20%). Statistically significant smaller copy number alterations (3.9 kb to 644 kb) were identified using STAC algorithm, including losses of *FGFR3, RECQL4*, *NOTCH1, PTEN, TSC2*, and/or *ASPSCR1* and gains of *ETV1*and/or *MAF*. Correlation analysis between genetic data and pathological data showed that gain of 1q21.1-q23.2 and gain of 8q11.1q13.1 are significantly associated with grade 2–4 and moderately or poorly differentiated HCCs, and gain of chromosome 5q was significantly associated with HCCs with vascular invasion, while gain of chromosome 7q is significantly associated with stage I HCCs.

**Conclusions:**

This study has provided a detailed map of genomic aberrations occurring in HCV-associated HCC and has suggested candidate genes. In addition, gene enrichment analysis on the recurrent abnormal regions indicated NF- kappaB and BMP signaling pathways in HCC development and progression. This study demonstrated that genomic microarray test can be used to distinguish HCC from non- neoplastic cirrhotic nodules and to identify prognostic factors associated with HCC progression using pathologically characterized FFPE samples. Our data support the utility of genomic microarray test for the diagnosis, risk stratification, and pathogenic studies of HCC.

## Background

Hepatocellular carcinoma (HCC) is one of the most common malignant neoplasm and represents the third leading cause of cancer-related death worldwide [1,2]. The incidence of HCC is increasing in the United States and Europe, mostly because of the high prevalence of hepatitis C virus (HCV) infection [3,4]. The molecular mechanism of a malignant transformation of hepatocyte induced by HCV infection is still largely unclear. The lack of good cellular and animal models of HCV hepatocarcinogenesis further hampers the understanding of the underlying mechanisms. Given that HCV is an RNA virus which replicates in the cytoplasm and has little potential for integration of its genome into host DNA [5,6], liver cirrhosis has generally been considered a prerequisite for HCV-infected livers to develop HCC. The pathogenesis of HCC in chronic HCV infection is generally accepted as chronic inflammation and injury, which leads to fibrosis with eventual progression to cirrhosis and subsequent development of HCC [7]. There are several clinical and pathological staging systems for HCC, but none of them include biological parameters as predictors for prognosis [8], and there has not been a standardized molecular classification of HCC. The low efficacy of systemic chemotherapies for HCC (<40%) [7] encourage intensive investigation to identify the molecular mechanisms implicated in the carcinogenesis of HCV associated HCC, an area of great need.

This study aimed to determine the patterns of recurrent genetic alterations and common pathways involved in the development and progression of HCV-associated HCC using high resolution genomic microarray analysis and the correlations of genetic alterations with tumor phenotype, clinical presentation and outcome to improve the identification of risk factors in molecular HCC subtypes. Although genomic studies for characterization of DNA copy number alterations of HCV-associated HCC [9,10] or HCC due to various or unspecified etiologies [11-14] have been conducted, these studies used either conventional comparative genome hybridization (CGH) on metaphase cells or low density Bacterial Artificial Chromosome (BAC) clone array-based CGH analysis, therefore, submicroscopic and small copy number alterations could be undetected. In addition, no correlation analysis between genetic alterations and clinical and pathological data of HCC was conducted to define clinically relevant subtypes for prognosis. Furthermore, all the specimens used in this study were formalin-fixed paraffin-embedded (FFPE) HCC tissues, demonstrating the feasibility of whole genome microarray analysis using FFPE HCC specimens, and paired non-neoplastic cirrhotic nodules were also examined for comparison.

## Results

### Clinical and pathologic characterizations of HCC specimens

A total of 15 cases from which the paraffin blocks contained enough tumors for DNA isolation were included. The patients’ demographics, clinical and pathologic features of the specimens are summarized in Table 1. The age of these patients ranged from 45 to 67 years with an average of 56 years. The tumor size ranged from 1 to 4 cm with an average of 2.2 cm. Pathological examinations of the background livers in the explanted specimens showed that all the cases had cirrhosis. All 15 cases were negative for hepatitis B co-infection by serology or viral DNA test, and occult hepatitis B was also excluded by PCR. Due to the retrospective nature of the study, only a subset of these specimens has HCV genotype data.

**Table 1:** **Summary of the patient demographics, clinical and pathologic features of the HCC specimens**

**HCC specimens**	**Age**	**Gender**	**Ethnicity**	**Etiology**	**HCV genotype**	**Cirrhotic liver**	**Grade**	**Tumor differentiation**	**Tumor stage (TNM)**	**Tumor size (cm)**	**Vascular invasion**
HCC01	45	m	caucasian	HCV	na*	yes	G2	moderate	T1NxMx	1.3	no
HCC02	57	m	Asian	HCV	na	yes	G4	poor	T1NxMx	2.7	no
HCC03	57	m	caucasian	HCV	na	yes	G2	moderate	T1NxMx	2	no
HCC04	49	m	unknown	HCV	na	yes	G4	poor	T3NxMx	3	yes
HCC05	65	m	unknown	HCV	2a	yes	G3	moderate	T2N0Mx	2.4	no
HCC06	50	m	caucasian	HCV	na	yes	G3	moderate	T2N0Mx	2.5	yes
HCC07	56	m	caucasian	HCV	2b	yes	G1	well	T1N0Mx	1.5	no
HCC08	62	m	caucasian	HCV	na	yes	G2	moderate	T2NxMx	2.1	no
HCC09	51	m	caucasian	HCV	1a	yes	G1	well	T1NxMx	1.8	no
HCC10	60	m	caucasian	HCV	3	yes	G3	moderate	T1NxMx	1.7	yes
HCC11	53	m	caucasian	HCV	na	yes	G3	moderate	T2NxMx	3	no
HCC12	57	m	unknown	HCV	na	yes	G1	well	T1N0Mx	2.3	yes
HCC13	48	m	Asian	HCV	na	yes	G2	moderate	T1NxMx	1	no
HCC14	66	m	Native American	HCV	na	yes	G1	well	T1NxMx	3	no
HCC15	51	m	caucasian	HCV	1a	yes	G1	well	T1NxMx	1.5	no

*na - not available.

### Whole genome microarray analysis using FFPE specimens

**Figure 1:**
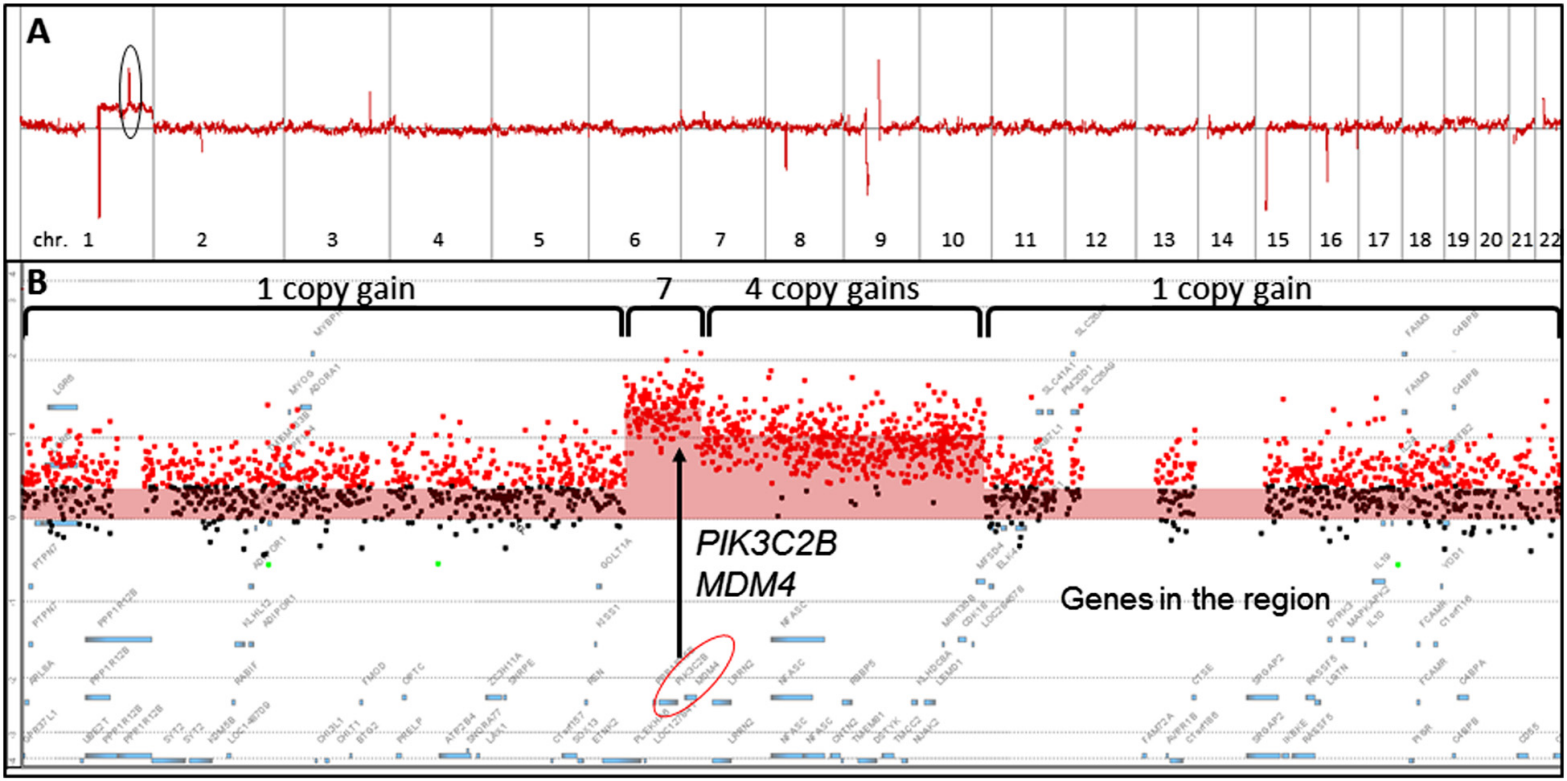
**Whole genomic profile of HCC using formalin-fixed paraffin-embedded (FFPE) specimen. A**. Genomic profile of HCV associated HCC (HCC01, Table 1), and the zoomed in area is in the black circle. **B**. Amplified gene regions on 1q32.1 with one, four or seven copy gains. Gene locations of *PIK3C2B* and *MDM4* are indicated with a circle*.* X-axis represents genomic intervals of chromosomes 1–22 (1**A**) or genes in amplified regions (1**B**). Y-axis represents log_2_Ratios.

DNA was extracted from FFPE tissues of 16 HCCs and 10 paired non-neoplastic cirrhotic liver tissues. Except for one HCC specimen, microarray analysis for copy number changes were successful for 15 HCC specimens and 10 paired non-neoplastic cirrhotic liver tissues, giving a 96.2% successful rate for microarray analysis using FFPE specimens. As an example, HCC01 showed a copy gain of the long arm of chromosome 1 (1q) (Table 1, Figure 1A,) and amplified regions containing multiple genes on 1q (Figure 1B). Tumor cellularity, the relative proportion of tumor and normal cells in a sample, affects the sensitivity of copy number detection, and can be estimated based on the review of H & E stained slide by the pathologist. In addition, the log2 ratio and copy number line fit plots by Cytogenomics can also be used to indicate clonal diversity and estimate tumor cellularity for each clone. For instance, the tumor cellularity in HCC01 was estimated to be approximately 57% based on the pathologist’s review of the H & E stained slide and the log2 ratio and copy number line fit plot by Cytogenomics. In addition, no clonal diversity in HCC01 was indicated by the log2 ratio and copy number line fit plot. Based on the values of log2 ratios and 57% tumor cellularity, the numbers of copy gains in the amplified regions on 1q of HCC01 were estimated to be four and seven copies (including gains of *MDM4* and *PIK3C2B*), respectively (Figure 1B).

### Recurrent copy number alterations in HCC

**Figure 2:**
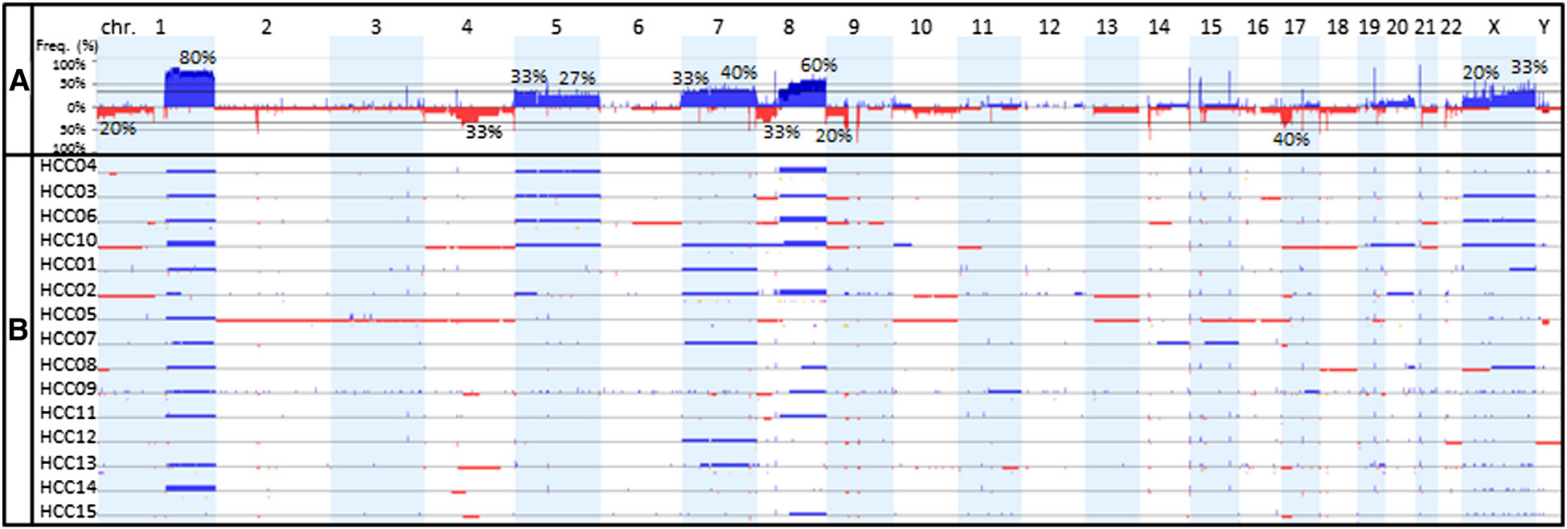
**Genomic profile of HCC specimens showing recurrent chromosomal gains and losses. A**. The accumulative frequency (or aggregate) plot for the HCC specimens with rate (%) labeled for the most frequent recurrent clonal chromosomal gains and losses in 15 HCV-associated HCC specimens. Blue bars indicate gains and red bars indicate losses. **B**. Genomic profiles of copy number alterations in individual HCC specimens.

Clonal chromosomal abnormalities were detected in all HCC samples but not in their paired non-neoplastic tissues. Accumulative and individual chromosomal imbalances in the HCV-HCC genomes are summarized in Figure 2. Recurrent copy gains and losses of genomic regions that were larger than 5 Mb in the HCV-HCC specimens were summarized in Table 2. The common chromosomal aberrations (>5 Mb) observed in HCC were chromosomal gains of 1q (80%), 8q (60%), 7q (40%), 5p (33%), 7p (33%), Xq (33%), 5q (27%), and Xp (20%), as well as chromosome losses of 17p (40%), 4q21.21-q26 (33%), 8p (33%), 1p36.11-pter (20%), and 9p (20%). The numbers of chromosomal imbalance larger than 5 Mb in each case ranged from 2 to 12 copy number changes per case with 7 copy number changes per case on average. No chromosomal aberrations were observed in the 10 paired non-neoplastic cirrhotic liver tissues that were available for examination.

**Table 2:** **Summary of regions of recurrent chromosome imbalance (>5 Mb), frequencies, and Sanger Census cancer genes in the region**

**Region**	**Region length (kb)**	**Cytoband location**	**Copy number**	**Frequency (%)**	**Number of genes**	**Number of sanger census cancer gene**	**Sanger census cancer gene**
chr1:732,712-24,356,209	23,624	p36.33 - p36.11	Loss	20.00	375	7	TNFRSF14, PRDM16, RPL22, CAMTA1, SDHB, PAX7, MDS2
chr1:143,582,356-249,250,621	105,668	q21.1 - q44	Gain	80.00	1155	17	PDE4DIP, BCL9, ARNT, TPM3, MUC1, PRCC, NTRK1, SDHC, FCGR2B, PBX1, ABL2, TPR, MDM4, ELK4, SLC45A3, H3F3A, FH
chr4:80,505,114-115,297,840	34,793	q21.21 - q26	Loss	33.33	167	2	RAP1GDS1, TET2
chr5:0–46,279,735	46,280	p15.33 - p11	Gain	33.33	181	2	IL7R, LIFR
chr5:49,584,189-180,704,505	131,120	q11.1 - q35.3	Gain	26.67	846	11	IL6ST, PIK3R1, APC, PDGFRB, CD74, ITK, EBF1, RANBP17, TLX3, NPM1, NSD1
chr7:0–57,675,692	57,676	p22.3 - p11.2	Gain	33.33	381	10	CARD11, PMS2, ETV1, HNRNPA2B1, HOXA9, HOXA11, HOXA13, JAZF1, IKZF1, EGFR
chr7:61,750,617-159,138,663	97,388	q11.21-q36.3	Gain	40.00	757	12	SBDS, ELN, HIP1, AKAP9, CDK6, MET, SMO, CREB3L2, KIAA1549, BRAF, EZH2, MLL3
chr8:13,403,518-29,636,237	16,233	p22 - p12	Loss	33.33	116	1	PCM1
chr8:46,967,935-145,730,376	98,762	q11.1 - q24.3	Gain	60.00	501	13	CHCHD7, TCEA1, PLAG1, NCOA2, NBS1, HEY1, CBFA2T1, UBR5, COX6C, EXT1, MYC, NDRG1, RECQL4
chr9:0–45,983,160	45,983	p24.3 - p11.2	Loss	20.00	278	6	JAK2, CD274, NFIB, MLLT3, FANCG, PAX5
chr17:3,728,554-12,900,807	9,172	p13.2 - p12	Loss	40.00	205	5	USP6, TP53, PER1, GAS7, MAP2K5
chr17:13,297,102-18,590,696	5,293,594	p12 - p11.2	Loss	33.33	77	0	
chrX:2,471,123-48,865,144	46,394	p22.33 - p11.23	Gain	20.00	257	7	ZRSR2, BCOR, KDM6A, SSX1, SSX4, WAS, GATA1
chrX:61,828,910-99,931,689	38,103	q11.1 - q22.1	Gain	26.67	156	4	MSN, MED12, NONO, ATRX
chrX:99,931,690-154,975,693	55,044	q22.1 - q28	Gain	33.33	613	5	SEPT6, ELF4, GPC3, PHF6, MTCP1

Statistically significant smaller copy number alterations were identified and ranged from 3.9 kb to 644 kb using STAC algorithm implemented in Nexus 7.5 (Table 3). Among the genes in these regions, known cancer genes based on the Sanger Census cancer gene list (http://cancer.sanger.ac.uk/cancergenome/projects/census/) included losses of *FGFR3, RECQL4, NOTCH1, PTEN, TSC2*, and/or *ASPSCR1* and gains of *ETV1* and/or *MAF*. In addition, copy gain involving Androgen receptor (*AR*) were observed in five of 15 HCCs analyzed.

**Table 3:** **Summary of statistically significant smaller copy number alterations (p-value < = 0.05) and genes in the region**

**Region**	**Region length (kb)**	**Cytoband location**	**Copy number**	**Frequency (%)**	**P-value**	**Genes**	**Gene symbols**	**Sanger census cancer gene**
chr1:142,898,654-143,481,059	582	q21.1	Loss	26.67	<0.001	0	has-mir-3118-2, has-mir-3118-2	
chr2:127,807,016-127,821,014	14	q14.3	Loss	20.00	0.021	1	BIN1	
chr2:141,745,577-141,790,622	45	q22.1	Gain	20.00	0.005	1	LRP1B	
chr2:236,033,001-236,677,413	644	q37.2	Gain	20.00	0.005	1	AGAP1	
chr3:196,726,226-196,756,289	30	q29	Loss	20.00	0.002	2	MFI2, MIF2-AS1	
chr4:1,792,487-1,809,740	17	p16.3	Loss	33.33	<0.001	1	FGFR3	FGFR3
chr4:69,305,095-69,412,970	108	q13.2	Gain	40.00	<0.001	2	TMPRSS11E, UGT2B17	
chr7:13,933,308-14,336,685	403	p21.2	Gain	46.67	<0.001	1	ETV1	ETV1
chr7:38,306,193-38,380,749	75	p14.1	Loss	33.33	<0.001	1	TARP	
chr7:156,798,142-156,928,704	131	q36.3	Loss	46.67	<0.001	2	MNX1, LOC645249	
chr8:145,737,034-145,741,006	4	q24.3	Loss	40.00	<0.001	1	RECQL4	RECQL4
chr9:139,390,677-139,425,667	35	q34.3	Loss	26.67	0.003	1	NOTCH1	NOTCH1
chr10:3,514,976-3,988,938	474	p15.2 - p15.1	Gain	26.67	0.008	1	KLF6	
chr10:89,581,346-89,717,798	136	q23.31	Loss	20.00	0.021	3	CFL1P1, KLLN, PTEN	PTEN
chr11:65,263,143-65,279,263	16	q13.1	Gain	26.67	0.003	1	MALAT1	
chr16:2,104,905-2,135,150	30	p13.3	Loss	20.00	0.021	1	TSC2	TSC2
chr16:79,628,093-79,633,200	5	q23.2	Gain	20.00	0.003	1	MAF	MAF
chr16:89,550,964-89,780,717	229753	q24.3	Gain	20.00	0.003	12	ANKRD11, SPG7, SNORD68, RPL13, CPNE7, DPEP1, CHMP1A, C16orf55, CDK10, SPATA2L, VPS9D1, LOC100128881	
chr17:79,848,607-79,974,997	126390	q25.3	Loss	26.67	0.02	11	ALYREF, ANAPC11, NPB, PCYT2, SIRT7, MAFG, MAFG-AS1, PYCR1, MYADML2, NOTUM, ASPSCR1	ASPSCR1
chr18:14,827,524-15,293,288	465764	p11.21	Loss	53.33	<0.001	2	ANKRD30B, MIR3156-2	
chr19:42,402,083-42,413,495	11412	q13.2	Loss	40.00	<0.001	1	ARHGEF1	
chrX:66,252,360-66,809,186	556827	q12	Gain	33.33	<0.001	1	AR	

### Correlation of recurrent genetic alterations with clinical and pathologic data

**Figure 3:**
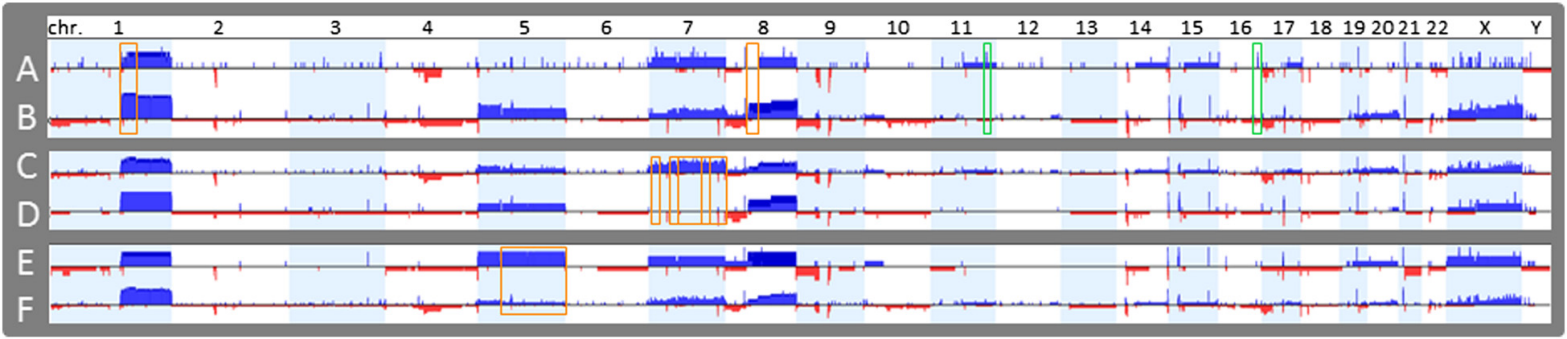
**Frequency difference plot for gains and losses between groups in the comparison analysis.** Comparison I: five of grade 1 and well differentiated HCCs (Figure 3 **A**) vs ten of grade 2, 3 or 4 and moderately or poorly differentiated HCCs (Figure 3 **B**). Comparison II: ten of Stage I HCC (Figure 3 **C**) vs five of Stage II or III HCCs (Figure 3 **D**). Comparison III: four of HCCs with vascular invasion (Figure 3 **E**) vs eleven of HCCs without vascular invasion (Figure 3 **F**). Regions of loss are indicated by red bars and regions of gains by blue bars.

Three comparison/correlation analyses were conducted and the difference in frequency for gains and losses in each group are shown in Figure 3 and significant genomic aberrations identified are summarized in Tables 4, 5 and 6. A subset of signature copy number alterations associated with specific HCC pathologic features was identified.

**Table 4:** **Result summary of the comparison analysis between grade 1/well differentiated HCCs and grade 2-4/moderately or poorly differentiated HCCs (Figure** 3 **A and B)**

**Region [hg19]**	**Cytoband location**	**Copy number**	**Region length (kb)**	**Grade 1 and well differentiated HCC (freq. %)**	**Grade 2–4 and moderately and poorly differentiated HCC (freq. %)**	**p-value**	**Number of genes**	**Sanger census cancer genes**
chr1:146,319,961-159,188,358	q21.1 - q23.2	Gain	12,868	40	100	0.022	261	BCL9, ARNT, TPM3, MUC1, NTRK1
chr8:46,967,935-67,313,267	q11.1 - q13.1	Gain	20,345	0	60	0.044	76	CHCHD7, TCEA1, PLAG1
chr11:113,937,346-114,144,024	q23.2	Gain	207	60	0	0.022	1 (ZBTB16)	
chr16:79,628,093-79,633,200	q23.2	Gain	5	60	0	0.022	1	MAF

**Table 5:** **Result summary of the comparison analysis between Stage I HCC and Stage II or III HCCs (Figure** 3 **C and D)**

**Region [hg19]**	**Cytoband location**	**Copy number**	**Region length (kb)**	**Stage I (freq. %)**	**Stages II-III (freq. %)**	**p-value**	**Number of genes**	**Sanger census cancer genes**
chr7:13,933,308-14,289,034	p21.2	Gain	356	70	0	0.026	2	ETV1
chr7:54,910,410-55,228,621	p11.2	Gain	318	70	0	0.026	1	EGFR
chr7:61,750,617-159,138,663	q11.21-q36.3	Gain	97,388	60	0	0.044	757	SBDS, ELN, HIP1, AKAP9, CDK6, MET, SMO, CREB3L2, KIAA1549, BRAF, EZH2, MLL3
chr7:115,832,560-116,493,846	q31.2	Gain	661	70	0	0.026	4	MET

**Table 6:** **Result summary of the comparison analyses between HCCs with vascular invasion and without vascular invasion (Figure** 3 **E and F)**

**Region [hg19]**	**Cytoband location**	**Copy number**	**Region length (kb)**	**Vascular invasion (freq. %)**	**No vascular invasion (freq. %)**	**p-value**	**Number of genes**	**Sanger census cancer genes**
chr5:49,584,189-180,704,505	q11.1 - q35.3	Gain	131,120	75	9.091	0.033	846	IL6ST, PIK3R1, APC, PDGFRB, CD74, ITK, EBF1, RANBP17, TLX3, NPM1, NSD1

In comparison analysis I, five grade 1 and well differentiated HCCs (A, Figure 3) were compared with ten grade 2–4 and moderately or poorly differentiated HCCs (B, Figure 3). Significant copy number aberrations associated with grade 1 and well differentiated HCCs are gain of 11q23.2 containing *ZBTB16* and gain of 16q23.2 containing *MAF* (Table 4). Significant copy number aberrations associated with grade 2–4 and moderately or poorly differentiated HCCs are gain of 1q21.1-q23.2 containing 261 genes including 5 cancer genes *BCL9, ARNT, TPM3, MUC1*, and *NTRK1* and gain of 8q11.1q13.1 containing 76 genes including 3 cancer genes *TCEA1, PLAG1*, and *CHCHD7* (Table 4). In addition, gains of chromosomes 5 and X and loss of 9p were only found in grade 2–4 and moderately or poorly differentiated HCCs although they did not reach statistical significance (Figure 3A and B).

Gene enrichment analysis on significant common aberrations identified by the comparison analysis I showed that the genes that are significantly enriched in grade 2–4 and moderately or poorly differentiated HCCs were involved in positive regulation of NF-kappaB transcription factor activity, including gains of *S100A9, S100A12, S100A8, IL6R, NTRK1, AIM2*, and *NLRP3* on 1q, and in regulation of chemokine production, including gains of *DARC* on 1q and *SNAI2* on 8q.

Furthermore, the numbers of chromosomal imbalance larger than 5 Mb in each case ranged from 2 to 6 copy number changes per case (on average 3.8 copy number changes per case) for grade 1 and well differentiated tumors when compared with 3 to 12 copy number changes per case (on average 7.1 copy number changes per case) for grade 2–4 or moderate or poorly differentiated tumors. The copy number variants (CNV) burden in terms of average number of CNVs for each category is significantly different with p-value of 0.023 using student’s t-test.

**Figure 4:**
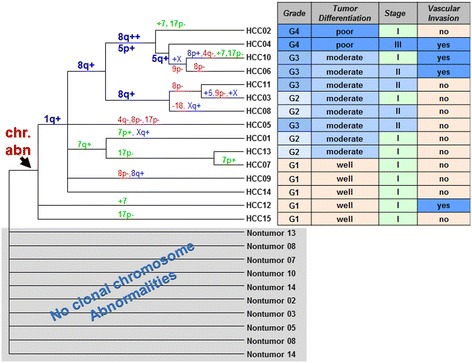
**The dendrogram generated from cluster analysis.** Genetic subgroups shown as clads/clusters were labeled with shared chromosomal aberrations on the branches leading to the clades or the HCC specimen. Chromosomal abnormalities partitioned with HCCs and formed a clad that was indicated by the arrow. Their paired non-neoplastic specimens were at the base of the dendrogram without clonal chromosomal aberrations detected. Clinical and pathological features associated with each HCC were listed in the table next to it.

In comparison analysis II, ten stage I HCCs (C, Figure 3) were compared with five stage II or III HCCs (D, Figure 3). Significant copy number aberrations associated with stage I tumors are gain of the long arm of chromosome 7 (7q), gain of 7p11.2 containing *EGFR*, and gain of 7p21.2 containing *ETV1* (Table 5). Among the genes in the significantly enriched in stage I on chromosome 7q are genes involved in negative regulation of hydrogen peroxide-mediated programmed cell death including *HGF* and *MET*, and genes in androgen metabolic process including *CYP3A4, AKR1D1*, and *SHH*, and mismatch repair complex and single strand binding protein gene *PMS2P5* and P*MS2P1*, *MCM7* and *SSBP1*. In addition, loss of chromosome 17p containing gene *TP53* was found in five of ten stage I HCCs, including three HCCs with whole arm deletion of 17p and two HCCs with deletions of most part of 17p (17p11.2-p13.2 of 18.5 Mb and 17p11.2-pter of 18.3 Mb respectively). However, this association did not reach statistical significance (Figure 4). *TP53* deletion is commonly associated with poor prognosis in neoplasm; however, the follow-up information is not available for most of the patients in this cohort.

In comparison analysis III, four HCCs with vascular invasion (E, Figure 3) were compared with eleven HCCs without vascular invasion (F, Figure 3). Significant copy number aberrations associated with HCCs with vascular invasion are gain of chromosome 5q (Table 6). Among the genes in the significantly enriched in tumors with vascular invasion on 5q are genes involved in BMP signaling pathway including genes *FST, ZFYVE16, RGMB, SMAD5, EGR1*, and *NKX2-5* on chromosome 5q, and genes involved in gamma-catenin binding including *FER, APC*, and *CTNNA1* on chromosome 5q.

### Cluster analysis

The dendrogram was generated based on recurrent chromosome aberrations of HCCs to construct genetic subgroups which reflected their genetic similarities in clusters (groups) (Figure 4). Chromosomal abnormalities partitioned well with HCCs which formed a clad (indicated by the arrow in Figure 4) with their paired non-neoplastic specimens at the base of the dendrogram. In the HCC clad, grade 1 and well differentiated HCCs were distributed as basal branches, while most of higher grade and less differentiated HCCs formed more organized clusters. Gain of 1q (80%) was most common and found in all HCCs with grade 2–4 and three of 5 HCCs with grade 1. The cluster of HCCs with gains of 1q, 8q, and trisomy 5 were associated with higher grades (2–4) and moderate to poor tumor differentiation, which were consistent with the results of comparison analysis (Tables 4, 5 and 6).

## Discussion

In this study, our data demonstrated the feasibility of whole genome microarray analysis using FFPE samples of hepatocellular carcinoma, as all of specimens used in this study were FFPE samples, including HCCs and paired non-neoplastic cirrhotic liver tissues. Using FFPE specimens for molecular and genomic studies are technically challenging due to the chemical crosslinks and degradation of DNA and RNA in these samples. However, it is important to establish the methods for molecular characterization using genomic approaches with FFPE tissue samples as they make up a vast archive of pathologically well-characterized clinical samples and are an immense resource that can be used for conducting biomarker investigation.

Contrary to many previous copy number aberration studies of HCC using conventional CGH with 10–20 Mb resolution or BAC array with 1–2 Mb resolution for genetic characterization of HCCs with various or unknown etiologies (Table 7) [9-14], this study focuses on HCV associated HCCs using high density whole genome oligo microarray analysis which provides higher resolution (>2 kb) to facilitate gene discovery. In addition, the levels of amplifications involving known oncogenic genes are readily detected. The copy number amplification can also be calculated based on the values of log_2_ ratio and tumor cellularity, as in specimen HCC01 which had 7-copy amplification of *MDM4* and *PIK3C2B* (Figure 1). *MDM4* is known to contain a p53 binding domain at the N-terminus and a RING finger domain at the C-terminus, and has been shown to interact with *E2F1* [15], *MDM2* [16,17] and P53 tumor suppressor protein [18], and overexpress in a variety of human cancers. *PIK3C2B* belongs to the phosphoinositide 3-kinase (PI3K) family which play certain roles in signaling pathways involved in cell proliferation, oncogenic transformation, cell survival, cell migration, and intracellular protein trafficking. Co-amplification of the adjacent genes may provide an additional growth advantage in HCC. In addition, *PIK3C2B* may serve as a potential therapeutic target. The protein encoded by *PIK3C2B* was demonstrated to play an essential role in HCV propagation in human HCC cells, and knockdown of *PIK3C2B* abolished HCV propagation in the cell [19].

**Table 7:** **Comparison of frequencies of major gains and losses of genomic regions from this study and previous reports**

**Method**	**Etiology**	**Chromosomal gain (frequency, %)**								**Chromosomal loss (frequency, %)**						**References**
		**1q**	**5p**	**5q**	**7p**	**7q**	**8q**	**17q**	**20q**	**1p**	**4q**	**6q**	**8p**	**9p**	**17p**	
Conventional CGH	HCV, HBV, or non-viral	46	27	<5	<5	<5	69	46	31	35	42	15	58	27	31	[12]
BAC or Oligo CGH	HCV, HBV, or non-viral	>25	NA*	NA	NA	NA	>25	>25	NA	NA	>25	>25	>25	>25	>25	[11]
Conventional CGH	HCV, HBV, or non-viral	49	<5	6	13	15	55	13	12	18	33	33	55	13	22	[13]
Conventional CGH	HCV or HBV	46	<5	<5	<5	<5	41	37	<5	24	39	61	44	24	<5	[14]
Conventional CGH	HCV only	46	5	5	9	9	31	43	27	37	48	23	28	9	37	[10]
Conventional CGH	HCV only	79	16	11	16	11	37	16	11	32	53	21	32	11	79	[9]
oligo CGH	HCV only	80	33	27	33	40	60	<5	<5	20	33	7	33	20	40	This study

*NA - note available.

Clonal chromosomal abnormalities were detected in all HCC samples but not found in their paired non-neoplastic tissues (Figure 4), demonstrating that chromosomal copy number aberrations detected by whole genome microarray analysis were tumor-associated somatic changes and may serve as good genetic markers to distinguish tumors from non-neoplastic cirrhotic nodules.

The frequent chromosomal aberrations (>5 Mb) found in this study, including gains of 1q and 8q and loss of 1p, 4q, 8p, 9p, and 17p, were largely consistent with previous reports for HCCs with various etiologies, including virus-associated and non-viral HCCs (Table 7) [9-14]. Similar pattern of chromosomal imbalances with different etiology suggests a common basic state for HCC development, most likely the chronic process of cirrhosis due to non-specific inflammatory and regenerative processes. However, frequent gains of chromosomes 5 (33%) and/or 7 (33-40%) found in this cohort (Table 2, Figures 2 and 4) have not been reported previously as common chromosomal aberrations in HCC (Table 7), suggesting that trisomy 5 and/or trisomy 7 could be the specific aberrations for HCV associated HCCs and future studies are warranted.

In addition, smaller deletions (3.9 – 582 kb) were identified to be statistically significant in this HCV-HCC cohort. These deletions contained known cancer genes based on the Sanger Census cancer gene list, including *FGFR3, RECQL4, NOTCH1, PTEN, TSC2,* and/or *ASPSCR1* (Table 3) which suggested their roles as tumor suppressor genes in the development of HCC. These genetic alterations were undetected by previous studies with conventional CGH and BAC arrays, most likely due to the low resolution of the analyses.

*FGFR3* appears to have dual actions in cancers. *FGFR3* can have both tumor suppressive and oncogenic properties. It was shown that *FGFR3* signal can limit tumor growth with epithelial origin. Therefore, for tumors of epithelial origin, loss of *FGFR3* was found in higher grade tumor while activating mutations of *FGFR3* were found in benign or low grade tumor with good prognosis [20]. Although little is known on *FGFR3* actions in HCC, *FGFR3* deletion was found mostly in grade 2–3 HCC in this cohort, indicating *FGFR3* as a tumor suppressor gene in HCC and is associated with HCC of higher grade. *FGFR3* oncogenic property is crucial for targeted therapy involving specific tyrosine kinase inhibitors. Loss of *NOTCH1* resulted in a continuous proliferation of hepatocytes and nodular regenerative hyperplasia in conditional *NOTCH1* knockout mouse model [21]. *PTEN* as a tumor suppressor, negatively regulating AKT/PKB signaling pathway by preferentially dephosphorylates phosphoinositide substrates, is mutated in a large number of cancers including 40-50% of human liver cancers such as HCC and cholangiocarcinoma [22]. *TSC2* is a tumor suppressor and is able to stimulate specific GTPases. Loss of *TSC2* leads to activation of MTOR and downstream signaling elements, causes endoplasmic reticulum (ER) stress, activates the unfolded protein response, and results in tumor development [23]. *TSC2* deletions were found in HCV-associated HCC with grade 2–3 and moderate differentiation and without vascular invasion in this cohort. This finding is consistent with the finding that decreased *TSC2* expression was found to be significantly correlated with higher grade and poor prognosis, but is inconsistent with the association with vascular invasion in a recent study [24]. The discrepancy may be explained by the population difference as all HCC cases are soly HCV associated in this study while the majority cases in Huang’s study were HBV associated HCCs with only two HCV-associated HCCs [24]. *RECQL4,* a DNA helicase that belongs to the RecQ helicase family, has not been previously recognized to be involved in HCC development. This study also showed that deletions involving whole gene *ASPSCR1* (alveolar soft part sarcoma chromosome region, candidate 1) were detected in HCC with statistical significance. The deletions of *ASPSCR1* most likely resulted in the loss of function and decreased expression of *ASPSCR1,* suggesting its role as a potential tumor suppressor gene in the development of HCC. In addition, loss of function and decreased expression of *ASPSCR1* has also been implicated in synthetic lethal interactions in cancer [25]. Gene *ASPSCR1* is relatively uncharacterized. An *ASPSCR1–TFE3* fusion protein due to an unbalanced translocation der(17) t(X;17) has been associated with alveolar soft-part sarcoma (ASPS), which resulted in unregulated transcription of *TFE3* and *TFE3-*regulated genes and a truncated allele of *ASPSCR1* with loss of function [26]. Future studies of the role of *ASPSCR1* in HCC are warranted.

There is great interest in identifying genetic markers of HCC that qualify for risk stratification. Most previous studies did not have detailed pathological data and correlation analysis between the genetics data and pathological data have not been conducted. We found several correlations between genetic data and clinicopathological data by comparison analysis. Gain of 1q21.1-q23.2 and gain of 8q11.1q13.1 were significantly associated with grade 2–4 and moderately or poorly differentiated HCCs (Table 4), including genes that are significantly enriched in positive regulation of nuclear factor-kappa B (NF-kappaB) transcription factor activity and regulation of chemokine production. This result suggests that NF- kappaB plays a role in the progression of HCC. The NF-κB transcription factor family is known to play an important role in many immune and inflammatory responses, and inflammation is considered a hallmark of cancer [27]. Enhanced expression of inflammatory cytokines and chemokines as key coordinators of the cross talk between hepatocytes and activated hepatic stellate cells was shown to be crucial in HCC development and progression, either by direct signaling or by recruiting immune cells [27,28] .

Comparison analysis also showed that gain of chromosome 5q was significantly associated with HCCs with vascular invasion (Table 6), which is a poor prognostic indicator for tumor spread. Gene enrichment analysis detected genes in bone morphogenetic proteins (BMP) signaling pathway including genes *FST, ZFYVE16, RGMB, SMAD5, EGR1*, and *NKX2-5* on chromosome 5q and in gamma-catenin binding including *FER, APC*, and *CTNNA1* on chromosome 5q (Table 6), suggesting that copy number gain and increased expression of BMP signaling may contribute to tumor progression and invasion. BMPs comprising the largest family within the TGF-β superfamily, originally reported as factors that induce bone and cartilage formation and development, have been shown to be critical for cancer development and progression [29]. Elevated expression levels of BMPs have been detected in many types of solid tumors, and BMP signaling pathway are intimately involved in both the inhibition and promotion of cancer progression [30]. A similar dual role for the superfamily member TGFβ is known to act as a tumor suppressor during the initial steps of tumorigenesis, but later found to promote tumor progression and invasion [31].

Furthermore, copy number variant (CNV) burden in terms of number of observed acquired CNV events (>5 Mb) in each case was greater in HCCs with grade 2–4 or moderate or poorly differentiated tumors (average 7.1 CNVs per case) compared to HCCs with grade 1 and well differentiated tumors (average 3.8 CNVs per case), indicating higher genomic instability in more advanced HCCs.

HCC has gender disparity with an increased frequency in males. Copy gain involving androgen receptor (*AR*) were observed in five HCCs of male patients in this cohort, including gain of Xq in four HCCs, and a gain containing only gene *AR* in one HCC. In addition, a gain containing the promoter region and first two exons of *AR* was observed in one HCC. It has been proposed that higher activity of androgen pathway functions as a tumor-promoting factor in male hepatocarcinogenesis, as knockout of *AR* expression in hepatocytes delayed the development of N’,N’-diethylnitrosamine (DEN)-induced HCC [32].

## Conclusions

This study has provided a detailed map of genomic aberrations occurring in HCV-associated HCC and has suggested candidate genes. As many frequent gains and losses are also common in HCCs with various etiologies, gains of chromosomes 5 and/or 7 appeared to be the specific aberrations for HCV-associated HCCs. While gain of 1q21.1-q23.2 and gain of 8q11.1q13.1 are significantly associated with grade 2–4 and moderately or poorly differentiated HCCs, gain of chromosome 7q is significantly associated with stage I HCCs, and gain of chromosome 5q was significantly associated with HCCs with vascular invasion which is poor prognosis indicator for tumor spreading. NF- kappaB and BMP signaling pathways were indicated for HCC development and progression. This study demonstrated that genomic microarray test can be used to distinguish HCC from non- neoplastic cirrhotic nodules and to identify signaling pathways involved in HCC development and prognostic factors associated with HCC progression using pathologically characterized FFPE samples. Our data support the utility of genomic microarray test for the diagnosis, risk stratification, and pathogenic studies of HCC.

## Methods

### FFPE tissue specimens

The pathological archives of representative number of hepatocellular carcinoma (HCC) were retrospectively reviewed and selected from the Department of Pathology at the University of Washington Medical center, including 15 HCV-associated HCC in cirrhotic livers, all from explanted liver specimens. The study protocol was reviewed and approved by the institutional review boards (University of Washington, Human Subjects Division). All specimens used in this study were formalin-fixed paraffin-embedded (FFPE) tissue specimens. Hematoxylin and eosin stained slides were reviewed to confirm the diagnosis and to grade and subclassify the HCC accordingly [33]. In addition, both cancer tissues and nearby cirrhotic non-neoplastic tissues were processed for the genetic characterization for 10 of 15 HCV-associated HCC cases, while in five of 15 cases, only HCC tissues were available for analysis.

We reviewed the clinical records of these patients and retrieved the data on demographic characteristics and clinical outcomes (Table 1). The tumor-node-metastasis (TNM) staging system of American Joint Committee on Cancer (Edition 7) was used to determine the T stage of the tumors [34].

### DNA extraction

Tumor and non-neoplastic areas were identified in H & E-stained slides and corresponding areas were dissected with a scalpel from the paraffin slides. Genomic DNA from FFPE tissue was extracted using manufacture recommended procedure (Agilent Technologies, Santa Clara, CA, USA) which is based on the method described by van Beers et al. [35] using the Qiagen DNeasy Blood & Tissue Kit (Qiagen Inc, Valencia, CA, USA). This procedure is optimized for 5 sections of 4–5 micron FFPE section containing about 1 cm^2^ of tissue. The concentration and the quality of genomic DNA were determined using Spectrophotometer NanoDrop ND-1000 (Thermo Fisher Scientific Inc., Wilmington, DE, USA).

### Genomic microarray analysis

Purified genomic DNA and the normal control reference DNA were then digested with restriction enzymes, labeled separately with contrasting fluorescence, and competitively hybridized to the custom designed high density oligonucleotide microarray as specified by the manufacturer (Agilent Technologies, Santa Clara, CA, USA). Chromosomal microarray analysis was performed on genomic DNA using the Agilent SurePrint G3 Cancer CGH + SNP 4x180K Array, a cancer-specific CGH + SNP microarray designed by Cancer Genomics Consortium (CGC) (http://www.chem-agilent.com/pdf/5990-9183en_lo_CGH+SNP_Cancer.pdf). Arrays were scanned using a DNA Microarray Scanner with SureScan High-Resolution technology (Agilent Technologies, Santa Clara, CA, USA). Whole genome microarray data were analyzed using Agilent CytoGenomics 2.5 to identify copy number changes. The global ADM2 algorithm with a threshold 6.0 and aberration filter for a minimum of five probes per region were applied. The CGH array data were also evaluated independently with second software Nexus Copy Number 7.5 (BioDiscovery, Inc. Hawthorne, CA, USA) to confirm the copy number changes identified by Cytogenomics. The log2R ratios provide information regarding copy number. These were determined by visual inspection. Genomic linear positions were given relative to NCBI build 37 (hg19, http://genome.ucsc.edu/). Analysis was limited to detect copy number changes that include at least 5 probes (markers) for deletions or duplications.

### Statistical analysis

To identify nonrandom gains and losses across multiple samples that are more likely to drive cancer pathogenesis, genetic aberrations in this cohort were evaluated for their statistically significance using statistical approaches with STAC algorithm adopted in Nexus 7.5 (BioDiscovery, Inc. Hawthorne, CA, USA). Significance Testing for Aberrant Copy number (STAC) is a method for testing the significance of DNA copy number aberrations across multiple microarray experiments to identify a set of aberrations that are aggregate in the overlapping regions such that it would not occur randomly [36]. The method applies two statistics, the frequency of aberration at a location across the entire sample set and p-value assigned to each location on the genome by using a multiple testing corrected permutation approach. The p-Value cut-off of 0.05 and the Aggregate % cut-off of 20% were used in the analyses.

Comparison analysis was conducted to correlate recurrent genetic alterations observed with clinical and pathologic data using Nexus 7.5 (BioDiscovery, Inc. Hawthorne, CA, USA). Statistical comparison using Fisher Exact test was performed to determine the p-value of certain genetic aberration in one group vs the other group in comparison. Scores exceeding the significant threshold with p-Value cut-off of 0.05 and below the differential threshold cut-off of 25% were used in the analyses. Comparisons between different groups of tumors based on the pathologic features were conducted to detect genomic regions that were significant different between the groups in a comparison based on the p-value. The factor sets compared in this study included tumor grade and differentiation, tumor stage, and vascular invasion.

Using significant common aberrations identified by comparison analysis, gene enrichment analysis on these selected regions of interest was conducted to attain the biological implications of these aberrations using Nexus 7.5 (BioDiscovery, Inc. Hawthorne, CA, USA). Enrichment analysis identifies gene ontology (GO) terms that are significantly overrepresented and identifies the genes annotated with these terms within this aberrant region [37,38].

### Clustering analysis

The genetic similarity between the tumor samples were evaluated using parsimony analyses by clustering similar recurrent aberration of genetic data using PAUP, version 3.1.1 [39,40] to generate a dendrogram that illustrated the arrangement of the clusters. The identified clusters with recurrent genetic aberrations were correlated with clinical and pathological features.
